# β-Subunit Binding Is Sufficient for Ligands to Open the Integrin α_IIb_β_3_ Headpiece*

**DOI:** 10.1074/jbc.M115.705624

**Published:** 2015-12-02

**Authors:** Fu-Yang Lin, Jianghai Zhu, Edward T. Eng, Nathan E. Hudson, Timothy A. Springer

**Affiliations:** From the Department of Biological Chemistry and Molecular Pharmacology, Program in Cellular and Molecular Medicine, Boston Children's Hospital, Harvard Medical School, Boston, Massachusetts 02115

**Keywords:** fibrinogen, hemostasis, integrin, platelet, receptor, allostery, conformation, structure

## Abstract

The platelet integrin α_IIb_β_3_ binds to a KQAGDV motif at the fibrinogen γ-chain C terminus and to RGD motifs present in loops in many extracellular matrix proteins. These ligands bind in a groove between the integrin α and β-subunits; the basic Lys or Arg side chain hydrogen bonds to the α_IIb_-subunit, and the acidic Asp side chain coordinates to a metal ion held by the β_3_-subunit. Ligand binding induces headpiece opening, with conformational change in the β-subunit. During this opening, RGD slides in the ligand-binding pocket toward α_IIb_, with movement of the βI-domain β1-α1 loop toward α_IIb_, enabling formation of direct, charged hydrogen bonds between the Arg side chain and α_IIb_. Here we test whether ligand interactions with β_3_ suffice for stable ligand binding and headpiece opening. We find that the AGDV tetrapeptide from KQAGDV binds to the α_IIb_β_3_ headpiece with affinity comparable with the RGDSP peptide from fibronectin. AGDV induced complete headpiece opening in solution as shown by increase in hydrodynamic radius. Soaking of AGDV into closed α_IIb_β_3_ headpiece crystals induced intermediate states similarly to RGDSP. AGDV has very little contact with the α-subunit. Furthermore, as measured by epitope exposure, AGDV, like the fibrinogen γ C-terminal peptide and RGD, caused integrin extension on the cell surface. Thus, pushing by the β_3_-subunit on Asp is sufficient for headpiece opening and ligand sliding, and no pulling by the α_IIb_ subunit on Arg is required.

## Introduction

**FIGURE 1.**
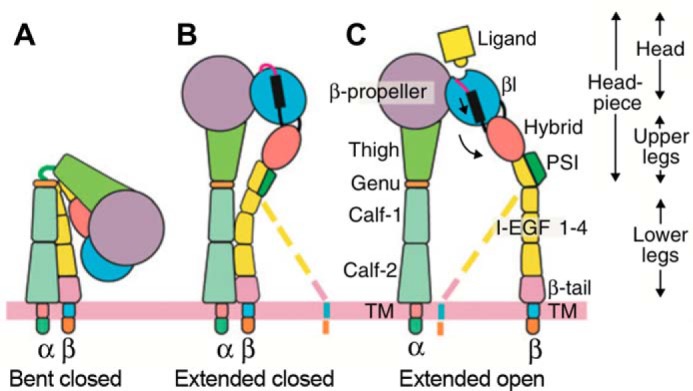
**Integrin domains and conformational changes.** Integrins are heterodimers of α- and β-subunits, each with large N-terminal extracellular domains, transmembrane domains (*TM*), and usually short C- terminal cytoplasmic domains. α_IIb_β_3_ can adopt three major conformations: bent with closed headpiece (*A*), extended with closed headpiece (*B*), and extended with open headpiece (*C*) (17, 23). *Colored dashed lines* emphasize the flexibility of the lower β-leg and lack of correlation of its position, in the absence of applied force, with whether the headpiece is open or closed. In integrin headpiece opening, downward pistoning of the α7-helix (*black bar*) of the βI domain is linked to swing out of the hybrid domain (*curved arrow*) and rearrangement of loops at the ligand-binding site. The open headpiece (*C*) has high affinity to ligands. *PSI* = plexin-semaphorin-integrin.

Integrins are heterodimeric adhesion receptors that transmit outside-in as well as inside-out signals across the cell membrane. Ligands of αI-less integrins such as α_IIb_β_3_ bind to a groove located in the interface between the α- and β-subunits (Fig. 1) (1). When activated, the ectodomain of α_IIb_β_3_ undergoes large-scale structural reshaping to an extended conformation with an open headpiece (Fig. 1) and binds with high affinity to the two distal ends of a fibrinogen dimer, causing platelets to form tight aggregates (2, 3). Arg-Gly-Asp (RGD)-binding integrins, including α_IIb_β_3_, α_v_β_3_, and α_5_β_1_, bind the Arg side chain to the β-propeller domain of the α-subunit via charged hydrogen bond(s). In contrast, the Asp side chain of RGD coordinates to Mg^2+^ held in the metal ion-dependent adhesion site (MIDAS)^3^ and forms multiple hydrogen bonds to NH backbone amide groups, including two in the β1-α1 loop of the β-subunit βI domain (3–5).

Within fibrinogen, α_IIb_β_3_ binds not to RGD, but to a ^400^HHLGGAKQAGDV^411^ sequence at the C terminus of the γ subunit (3, 6, 7). Lys-406 of γC forms charged hydrogen bonds with the α_IIb_ subunit; that is, Lys-406 of γC is functionally equivalent to the Arg of RGD. The side chain carboxyl group of Asp-410 directly coordinates to the β_3_-subunit MIDAS Mg^2+^ and forms hydrogen bonds to the βI β1-α1 loop backbone, equivalently to the Asp of RGD (3). Additionally, the C-terminal α-carboxyl group of γC Val-411 forms a water-mediated, indirect coordination to the Ca^2+^ held in the adjacent to MIDAS (ADMIDAS) (3).

We recently soaked different concentrations of RGD peptide into crystals containing the α_IIb_β_3_ headpiece in the closed conformation (assigned as state 1) and resolved six intermediate states (states 2–7) between the low-affinity closed headpiece and the high-affinity open headpiece (state 8) (4). Between states 1 and 8, the β1-α1 loop, which supplies three of the side chains that coordinate the MIDAS Mg^2+^ ion, moved toward the Asp side chain, enabling the Asp side chain to form hydrogen bonds to β1-α1 loop backbone NH groups. RGD slid in its binding groove, enabling its Arg side chain to closely approach α_IIb_ and to eventually form a charged hydrogen bond to α_IIb_ Asp-224. However, not until state 7 did a singular, well resolved electron density map appear for the Arg side chain. In states 1–6, the Arg side chain showed either weak density, reflecting low occupancy, or multiple conformations, including water-mediated hydrogen bonds to Asp-224. In contrast, the Asp of RGD always had good density and always directly coordinated with the MIDAS Mg^2+^ ion, suggesting that Asp binding to the β-subunit might be the main driver of headpiece opening. On the other hand, it was also possible that the attraction of the Arg side chain to the oppositely charged Asp-224 in α_IIb_ was responsible for opening by pulling RGD, and with it the βI domain β1-α1 loop, toward α_IIb_. Headpiece opening, rather than extension, is what greatly increases (by >100-fold) integrin affinity for ligands (4, 8). Headpiece opening is communicated across the βI domain by α7-helix pistoning to swing out of the hybrid domain (Fig. 1), which is a large-scale conformational change capable of being transmitted through the long integrin ectodomain legs to the cytoplasmic domains (1). Therefore, the question of whether headpiece opening is intrinsic to the β-subunit, or requires a pull by the α-subunit, is key to understanding both the biology and the mechanochemistry of integrins.

To resolve this issue, we have examined binding to α_IIb_β_3_ of truncated fibrinogen γC AGDV peptides that lack the Lys of KQAGDV, and thus cannot bind to the α_IIb_ subunit. Previous studies have shown that the QAGDV pentapeptide blocks binding of full-length fibrinogen to human platelets (9). Furthermore, CHO K1 cells transfected with α_IIb_β_3_ could adhere to monolayers functionalized with AGD or RGD peptides equally well, and adhesion to AGDVC monolayers was inhibited by similar concentrations of RGDS and AGDV peptides (10). Our studies address multiple questions beyond the minimal requirements for integrin headpiece opening. These include how AGDV peptide can bind and activate, and the specific role of its C-terminal carboxyl group in interaction with the ADMIDAS.

Remarkably, we find comparable affinities of AGDV peptide, fibrinogen γC dodecapeptide, and RGD peptide for the α_IIb_β_3_ integrin headpiece. AGDV can induce complete headpiece opening in solution, and crystals soaked with AGDV for varying durations defined the structural basis for AGDV-induced headpiece opening. Moreover, the C-terminal carboxyl of AGDV contributes to affinity by a highly geometrically constrained and hence highly specific water-mediated interaction with the ADMIDAS metal ion.

## Experimental Procedures

### 

#### 

##### Ligands

FITC-aminohexanoyl-HHLGGAKQAGDV (FITC-dodecapeptide) was synthesized by GenScript (Piscataway, NJ). Unlabeled fibrinogen γC dodecapeptide was from American Peptide Company (Sunnyvale, CA). All other peptides were synthesized by GenScript with >95% purity. Unless otherwise specified, peptides were not modified at either terminus. Ro-435054 was a generous gift from Dr. Paul Gillespie at Roche.

##### Recombinant α_IIb_β_3_ Headpiece

Expression and purification of α_IIb_β_3_ headpiece were as described (11).

##### Fluorescence Anisotropy Binding Assay

Binding affinities for different peptide ligands were measured using fluorescence anisotropy as described previously (12). Anisotropy is defined as (*F*‖ − *F*⊥)/(*F*‖ + 2*F*⊥), where *F*‖ and *F*⊥are the fluorescence intensities parallel and perpendicular to the excitation plane, respectively. mA units (shown in figures) correspond to 1,000 × anisotropy. The affinity of the FITC-dodecapeptide probe (used at 3 nm) for the α_IIb_β_3_ headpiece (*K*_*D*_^F*^) was measured by increasing the concentration of purified recombinant α_IIb_β_3_ headpiece to saturation. *K*_*D*_^F*^ was fitted using one-site, specific binding; *i.e. A* = *A*_max_ × *C*_itg_/(*K*_*D*_^F*^ + *C*_itg_), where *A* represents the anisotropy signal from specific binding, *C*_itg_ represents the concentration of integrin α_IIb_β_3_ headpiece, and *A*_max_ is the maximum anisotropy signal. Background anisotropy obtained by adding an excess of 10 μm tirofiban was subtracted from anisotropy.

After determining *K*_*D*_^F*^, unlabeled peptides were used to compete FITC-dodecapeptide probe (3 nm) binding to 300 nm α_IIb_β_3_ headpiece. I_50_ values for unlabeled peptides were obtained by least squares fitting. I_50_ values were converted to affinity values (*K*_*D*_^*p*^) for each peptide of interest (12) using

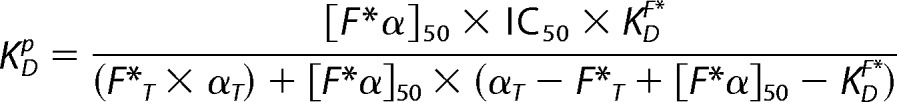
 where *K*_*D*_^F*^ is the FITC-dodecapeptide binding affinity to α_IIb_β_3_ headpiece (643 nm), F*_T_ is the total concentration of FITC-dodecapeptide (3 nm), α_T_ is the total concentration of α_IIb_β_3_ headpiece (300 nm), IC_50_ and I_50_ are the free and total concentrations of the peptide of interest causing the displacement of 50% of specifically bound FITC-dodecapeptide (*i.e.* IC_50_ = I_50_ − 0.5 × 300 nm), and [F*α]_50_ is the concentration of FITC-dodecapeptide bound to α_IIb_β_3_ headpiece at I_50_. All measurements were in 20 mm HEPES, pH 7.5, 150 mm NaCl, 2 mm MnCl_2_, 0.1 mm CaCl_2_. The Prism GraphPad program (La Jolla, CA) was used to fit the saturation binding curve, inhibition curves, and I_50_ values.

##### Size-exclusion Chromatography and Dynamic Light Scattering (DLS)

The α_IIb_β_3_ headpiece with coiled-coils was treated with chymotrypsin (chymotrypsin/integrin mass ratio = 1/200) at room temperature (13) for ∼16 h to remove C-terminal α-helical coiled-coils and to leave intact the thigh domain in the α-subunit. Digested headpiece was purified by passage through a nickel-nitrilotriacetic acid column (13). For Stokes radius measurements, the re-purified headpiece at 10 μm (1 mg/ml by *A*_280_) was incubated with 1 mm peptide ligand in 20 mm TBS with 1 mm Mg^2+^ and 1 mm Ca^2+^ at 25 °C for 30 min and then applied to a Superdex 200 column (GE Healthcare Life Sciences) pre-equilibrated with the same buffer containing 1 mm peptide. Calibration and conversion of elution volume to Stokes radius was as described (11). For DLS, 20 μm chymotrypsin-treated, re-purified headpiece was filtered through a 100 nm cutoff membrane (Millipore Ultrafree centrifugal filter), incubated with 1 mm peptide in TBS plus 1 mm Mg^2+^/Ca^2+^ for 10 min at 25 °C in the cuvette, and read in the Viscotek 802 DLS (Viscotek Corp.). The DLS-derived hydration radii (*R_h_*) were obtained by intensity-based fitting using the OmniSize program for 2–3 independent measurements using the same batch of integrin headpiece sample, but measured in different days. Each measurement was an average of at least fifteen 10-s reads.

##### Crystallization, Ligand Soaking, and Structure Determination

The α_IIb_β_3_ headpiece was crystallized in the closed conformation with the thigh domain removed (11). Briefly, chymotrypsin- and carboxypeptidase-treated α_IIb_β_3_-10E5 Fab complex was concentrated to 10 mg/ml in TBS (20 mm Tris, 150 mm NaCl, pH 7.4) with 1 mm Mg^2+^, 1 mm Ca^2+^ and crystallized in 11% PEG 8000, 0.2 m ammonium sulfate, 0.1 m Tris-HCl, pH 8.9 at 4 °C (11). Crystals were then stabilized with the same crystallization buffer but containing 15% PEG 8000, and glycerol concentration was incremented in 5% steps to 20% for cryoprotection. Finally, crystals were soaked in ligand in the same buffer with defined metal conditions for a specified duration at 4 °C (see Table 1). Diffraction data were collected at beamline ID-23 of the Advanced Photon Source (Argonne, IL). Resolution limit was determined by cross-correlation (14). Refinements using Phenix (15) were as described previously (4).

##### Ligand-induced Binding Site (LIBS) Epitope Expression

CHO K1 cells stably transfected with full-length α_IIb_β_3_ (16) were incubated at room temperature with serially diluted peptide in 20 mm HEPES, pH 7.4, 150 mm NaCl, 5.5 mm glucose, 1% BSA, 1 mm Mg^2+^/Ca^2+^, and 10 μg/ml LIBS1 antibody for 30 min. Mixtures were washed twice in the same buffer minus LIBS1. The washed cells were incubated with FITC-conjugated anti-mouse antibody at room temperature for 30 min, washed again, chilled on ice, and subjected to flow cytometry. The dose-response curves were fit, and EC_50_ values were calculated by Prism GraphPad.

## Results

### 

#### 

##### Affinities of Peptides Lacking Arg or Lys for the α_IIb_β_3_ Headpiece

**FIGURE 2.**
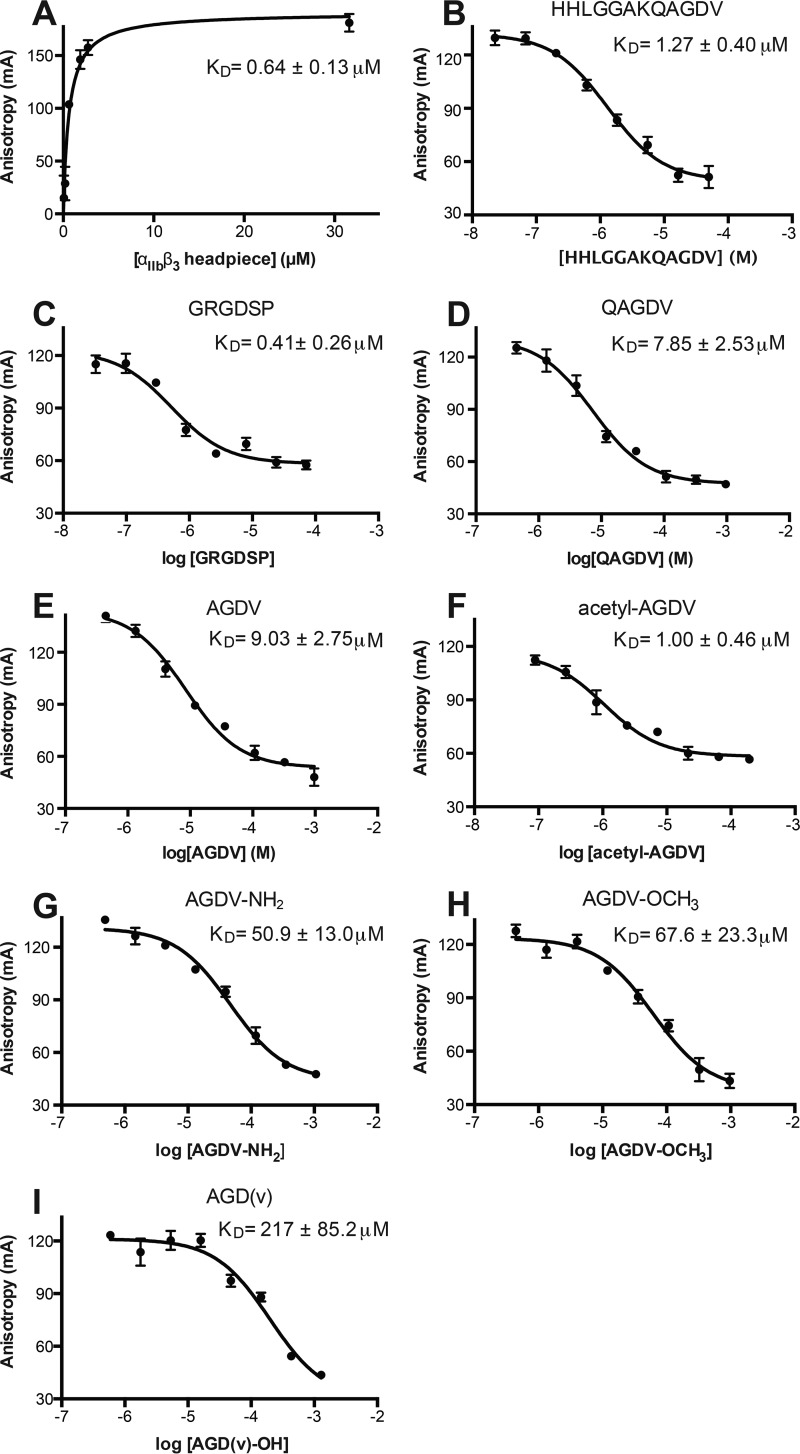
**Saturation binding, competition curves, and calculated *K_D_* values from fluorescence anisotropy.** *A*, saturation binding of FITC-dodecapeptide. *B–I*, competitive binding of the indicated peptides. The *K_D_* values were calculated from IC_50_ values as described under “Experimental Procedures.” *Error bars* indicate mean ± S.D.

Truncated fibrinogen γC peptides such as QAGDV and AGD have been shown to block fibrinogen binding to platelets with similar IC_50_ values of ∼100 μm (9), but more precise affinity measurements are required to understand the contribution of Lys or Arg. To test whether the Arg of RGD or Lys of KQAGDV is required for integrin binding, we quantitated the binding affinity to purified α_IIb_β_3_ headpiece in buffer with 2 mm Mn^2+^ and 0.1 mm Ca^2+^ using fluorescence polarization anisotropy. The fluorescent probe FITC-aminohexanoyl-HHLGGAKQAGDV bound saturably with a dissociation constant (*K_D_*) = 0.64 ± 0.13 μm (Fig. 2*A*). Competition with fluorescent probe at 3 nm and integrin at 300 nm was then used to determine the *K_D_* values of different peptides (Fig. 2, *B–F*) (see “Experimental Procedures”). The fibrinogen γC dodecapeptide and fibronectin hexapeptide GRGDSP showed *K_D_* of 1.27 ± 0.08 and 0.41 ± 0.26 μm, respectively (Fig. 2, *B* and *C*). In contrast, QAGDV and AGDV bound more weakly, with *K_D_* of 7.85 ± 2.53 and 9.03 ± 2.75 μm, respectively (Fig. 2, *D* and *E*). *N*-acetylated AGDV (Ac-AGDV) binds ∼9 times more strongly (*K_D_* = 1.00 ± 0.46 μm, Fig. 2*F*) than unmodified AGDV. Thus, Ac-AGDV has an affinity almost identical to the native γC dodecapeptide (Fig. 2, *B–F*).

##### AGDV Induces Complete Headpiece Opening in Solution

**FIGURE 3.**
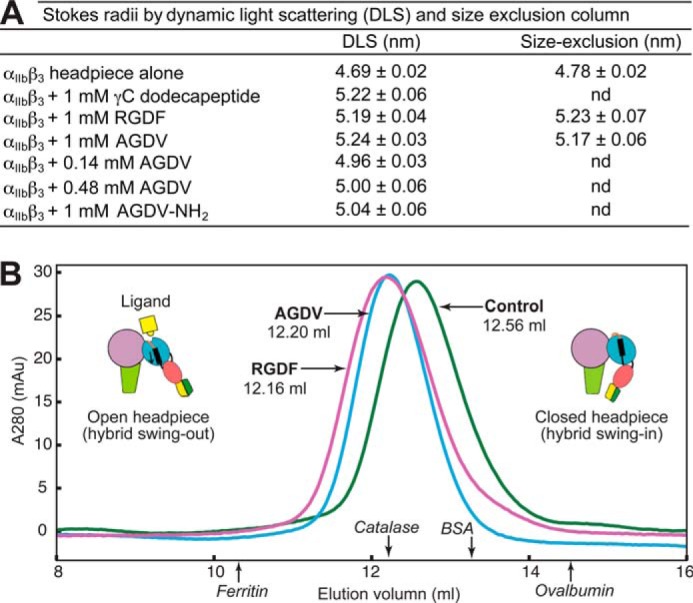
**Peptide ligands without Arg or Lys can induce full headpiece opening in Mg^2+^/Ca^2+^.** *A*, Stokes radii measured by DLS (data measured in triplicate, mean ± S.D.) and size exclusion chromatography (Superdex 200, values measured in duplicate, mean and difference from mean). *nd* = not determined. *B*, overlaid Superdex 200 chromatograms of unclasped αIIbβ3 headpiece in the absence (*green*) or presence of 1 mm RGDF peptide (*purple*) or 1 mm AGDV peptide (*blue*).

We determined whether headpiece opening can occur without the Arg of RGD or the Lys of fibrinogen γC peptide. AGDV-induced opening (*i.e.* hybrid domain swing-out, Fig. 1, *B* and *C*) of a six-domain α_IIb_β_3_ headpiece fragment was measured as an increase in Stokes radius (Fig. 3) (11). The hydrodynamic radii of closed and open six-domain α_IIb_β_3_ conformations were estimated from crystal structures (3, 17) with HYDROPRO (18) as 4.68 and 5.03 nm, respectively. When incubated with 1 mm dodecapeptide and RGDF peptide (a high-affinity ligand of α_IIb_β_3_(19)), in the resting condition (TBS, pH 7.4, plus 1 mm Mg^2+^ and Ca^2+^), the Stokes radius of α_IIb_β_3_ headpiece increased from 4.69 ± 0.02 to 5.22 ± 0.06 nm and 5.19 ± 0.04 nm, respectively (Fig. 3*A*) as measured by DLS. A similar increase (to 5.24 ± 0.03 nm) was observed with 1 mm AGDV peptide. The change in Stokes radius was also measured by size-exclusion chromatography (Fig. 3*B*). The elution volume shifted from 12.56 ± 0.01 ml in buffer alone to 12.15 ± 0.06 ml in the presence of 1 mm RGDF and to 12.19 ± 0.05 ml in AGDV. The corresponding Stokes radius shifted from 4.78 nm in buffer to 5.23 nm in RGDF and to 5.17 nm in AGDV (Fig. 3*A*). The DLS and gel-filtration results (Fig. 3*A*) demonstrated that a Lys or Arg residue is not essential for inducing full headpiece opening in α_IIb_β_3_, because at 1 mm peptide concentration, AGDV, dodecapeptide, and RGD caused virtually indistinguishable increases in Stokes radius.

##### Crystal Structures

**FIGURE 4.**
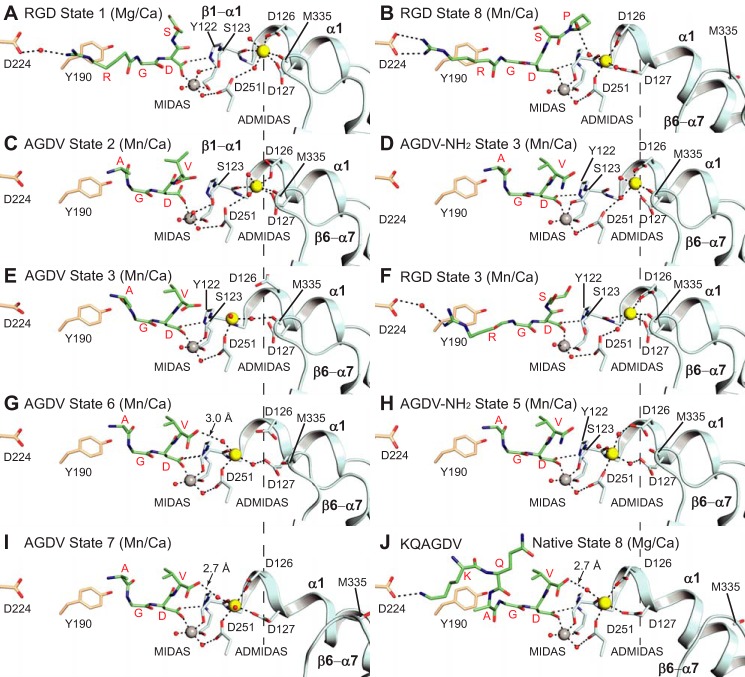
**Crystal structures.** Structures with soaked in AGDV and AGDV-NH_2_ determined here (*C–E* and *G–I*) are compared with previously determined structures (*A*, *B*, *F*, and *J*) (3, 4). Residues of the α_IIb_ and β_3_-subunits are shown with *wheat* and *cyan carbons*, respectively. MIDAS and ADMIDAS metals are shown as *gray* and *yellow spheres*, respectively. *Smaller red spheres* represent water molecules. *Black dashes* represent hydrogen bonds and metal ion coordinations.

We soaked AGDV peptide into α_IIb_β_3_ headpiece crystals in the closed conformation (state 1) in 1 mm Mn^2+^ and 0.2 mm Ca^2+^ and were able to trap four distinct intermediate states during headpiece opening (Table 1). Soaking times varied from 2 h to 1 week; the *t*_½_ value for diffusion of a ligand of comparable size (fluorescein) into crystals of size comparable with ours (0.2 × 0.05 × 0.01 mm) is 16 min (20). Two copies of the α_IIb_β_3_ headpiece complex (molecules 1 and 2) exist in one asymmetric unit (4), so we were able to obtain two different conformational states in a single crystal (Table 1). Based on the conformation and position of the moving parts of the βI domain, *i.e.* the β1-α1 loop, α1-helix, β6-α7 loop, and the ADMIDAS metal, each intermediate (Fig. 4) is assigned a state number between 1 (Fig. 4*A*) and 8 (Fig. 4*B*) corresponding to previous RGD-bound intermediates (4). AGDV binds in the groove formed between the α- and β-subunits (Fig. 4, *C*, *E*, *G*, and *I*), but has limited contact with α, because it occupies only a portion (about two-thirds) of the groove occupied by RGD (Fig. 4*B*). The N-terminal amine of AGDV is >9.0 Å away from Asp-224 of α_IIb_, which forms salt bridges to the Arg of RGD and the Lys of KQAGDV. Moreover, unlike the extended backbone conformation adopted by RGD (Fig. 4, *A* and *B*), the backbone of AGDV is curved (Fig. 4, *C*, *E*, *G*, and *I*).

**TABLE 1:** **X-ray diffraction and structure refinement statistics**

	Ligand			
	50 mm AGDV (Mn/Ca),*^a^* 1 week	50 mm AGDV (Mn/Ca),*^a^* 2 h	50 mm AGDV-NH_2_ (Mn/Ca),*^a^* 2 h	1 mm Ro-435054 (Mg/Ca),*^b^* 2 h
**Data collection*^c^***				
Space group	P2_1_2_1_2	P2_1_2_1_2	P2_1_2_1_2	P2_1_2_1_2
Unit cell				
*a*, *b*, *c* (Å)	256.9, 144.4, 104.6	259.9, 144.5, 104.7	259.4, 144.3, 104.6	259.3, 144.4, 105.0
α, β, γ (°)	90, 90, 90	90, 90, 90	90, 90, 90	90, 90, 90
Resolution (Å)	50–2.60 (2.64–2.60)	50–2.85 (2.90–2.85)	50–2.75 (2.80–2.75)	50–2.70 (2.77–2.70)
Completeness (%)	97.5 (73.9)	99.6 (94.0)	94.9 (87.9)	98.6 (87.0)
*R*_merge_	22.2 (427)	18.9 (362)	15.1 (208)	17.1 (243)
I/σ(I)	12.7 (0.4)	18.2 (0.3)	10.2 (0.6)	4.84 (0.4)
CC½	99.4 (18.6)	98.5 (12.9)	98.7 (18.9)	98.4 (10.8)

**Refinement*^c^***				
Resolution (Å)	50–2.60 (2.64–2.60)	50–2.85 (2.90–2.85)	50–2.75 (2.80–2.75)	50–2.70 (2.77–2.70)
No. of reflections	117,195 (5,179)	92,354 (5,041)	107,747 (10,641)	106,571 (9,580)
*R*_work_	0.230 (0.385)	0.227 (0.395)	0.188 (0.320)	0.212 (0.360)
*R*_free_	0.252 (0.425)	0.252 (0.414)	0.236 (0.334)	0.238 (0.362)
CC_work_	0.946 (0.466)	0.926 (0.258)	0.939 (0.377)	0.947 (0.357)
CC_free_	0.907 (0.229)	0.911 (0.384)	0.890 (0.595)	0.945 (0.492)
No. of non-hydrogen atoms				
Protein	20,771	20,791	20,878	20,835
Ligand/ion	50/14	50/14	50/14	72/13
Water	670	585	992	742
B factors				
Protein	96.5	117	97.3	92.5
Ligand/ion	72.0/101	101/134	78.0/116	72.0/52.4
Water	59.7	64.2	60.4	60.7
r.m.s.*^d^* deviations				
Bond lengths (Å)	0.003	0.005	0.004	0.005
Bond angles (°)	0.680	0.640	0.662	0.512
Molecules/asymmetric unit	2	2	2	2
Conformational states (molecule 1/molecule 2)	State 7/state 2	State 6/state 3	State 5/state 3	State 6/state 3
**PDB code**	**4Z7N**	**4Z7O**	**4Z7Q**	**5HDB**

*^a^* Mn/Ca: 2 mm MnCl_2_, 0.1 mm CaCl_2_.

*^b^* Mg/Ca: 1 mm MgCl_2_, 1 mm CaCl_2_.

*^c^* Numbers in parentheses correspond to the highest resolution shell.

*^d^* r.m.s., root mean square.

When compared with RGD (Fig. 4, *A* and *B*) and dodecapeptide structures in complex with the natively open headpiece in state 8 (Fig. 4*J*), the Φ angle of Gly of AGDV is rotated about 180° (Fig. 4, *C*, *E*, *G*, and *I*). This correlates with the markedly different backbone carbonyl oxygen orientation of the Arg in RGD (Fig. 4, *A*, *B*, and *F*) and Ala in KQAGDV (Fig. 4*J*) when compared with Ala in AGDV and AGDV-NH_2_ (Fig. 4, *C–E* and *G–I*). The peptide α-amino group, with a p*K_a_* estimated to be ∼8, is weakly basic, and would be partially charged at the binding assay pH of 7.4 and the crystallization pH of 8.9. To test for a weak π-cation interaction between the N-terminal primary amine and Tyr-190 of α_IIb_ (plan distance ∼4 Å), we abolished protonation by N-terminal acetylation. Acetylation increased peptide affinity (Fig. 2*F*) and therefore ruled out a contribution by a charged α-amino group to AGDV to binding.

Overall, the intermediate states caused by AGDV are similar to those induced by GRGDSPK (4) (Fig. 4). The exception is AGDV state 3, in which the β1-α1 loop backbone is shifted similarly to that with RGD in state 3, but the ADMIDAS Ca^2+^ ion is shifted more toward the MIDAS and has lost its coordinations to Asp-126 and Asp-127 (Fig. 4*E*). In contrast, the ADMIDAS Ca^2+^ ion retained its Asp-126 and Asp-127 coordinations in RGD state 3 (Fig. 4*F*). However, electron density for the ADMIDAS Ca^2+^ in AGDV state 3 is weak, which suggests an ill-defined position in shape-shifting. Importantly, during soaking for 1 week, AGDV induced the β_3_-subunit in molecule 1 in crystals to shift to state 7, *i.e.* almost all the way to the open conformation, despite little interaction with the α_IIb_ subunit (Fig. 4*I*).

##### The Pathway of AGDV-induced Headpiece Opening

The Asp carboxylate of AGDV in state 2 (Fig. 4*C*) shows a different angle from later states (Fig. 4, *E*, *G*, and *I*), with one carboxyl oxygen coordinating the MIDAS, and the other oxygen pointing away from the binding pocket. In this orientation, the ligand Asp would clash with the Tyr-122 side chain in the following states. In state 3, the Asp carboxyl re-orientates to enable for Tyr-122 and Ser-123 in the β1-α1 loop to move closer to the ligand and forms a hydrogen bond with the Tyr-122 backbone (Fig. 4*E*). Between states 2 and 3, the side chain hydroxyl of Ser-123 moves closer to the ligand and replaces a water molecule in the MIDAS to directly coordinate with the MIDAS metal ion (Fig. 4, *C–E*). The direct coordination between the Ser-123 side chain and the MIDAS and the hydrogen bond between the Asp of AGDV and the Tyr-122 backbone each remain in place through later states. However, in addition, between states 2 and 3, the valine side chain of AGDV switches to a different rotamer that is sustained through state 7, and the C-terminal carboxyl group rotates toward the ADMIDAS metal (compare Fig. 4, *E*, *G*, and *I* with Fig. 4*C*).

In state 6, the β1-α1 loop continues its movement toward the ligand (Fig. 4*G*). At this point, the Cβ carbon of Ser-123 is 2.4 Å away from its position in state 1. In state 6, the ADMIDAS metal ion is 3.4 Å away from its position in state 1 and is much closer to the C-terminal carboxyl of AGDV (4.8 Å). Importantly, starting from state 6, the C-terminal carboxyl of AGDV forms an indirect, water-mediated coordination with the ADMIDAS metal ion (Fig. 4*G*). Between states 6 and 7, the Cα atoms of Asp-126 and Asp-127 on the α1-helix move 2.2 Å toward the ADMIDAS metal ion (and 3.5 Å from state 1) and form direct coordinations (Fig. 4*I*). In addition, the β6-α7 loop flips away from the ADMIDAS to accommodate movement of the α1-helix toward the ligand (Fig. 4, *G* and *I*). In state 7, the conformations of the β1-α1 loop, α1-helix, and ADMIDAS metal of βI are very close to the natively open state 8 bound to fibrinogen γC dodecapeptide (compare Fig. 4*I* with Fig. 4*J*). The α-carboxyl of AGDV now forms a stronger 2.7 Å hydrogen bond with an ADMIDAS-coordinating water molecule (Fig. 4*I*).

##### The C-terminal Carboxyl Group Is Not Required for Headpiece Opening

We tested the role of the AGDV peptide α-carboxyl group in headpiece opening. We first measured the affinities of a series of C-terminally modified AGDV peptides, including AGDV-NH_2_ (amidated), AGDV-OCH_3_ (*O*-methylated), and AGD(v) (d-valine in place of l-valine). Both AGDV-NH_2_ and AGDV-OCH_3_ showed weaker binding than AGDV, with 5.6- and 7.5-fold reductions in affinity, respectively (Fig. 2, *G* and H), in agreement with results using amidated dodecapeptide (21). On the other hand, substituting l-valine with d-valine in AGD(v) resulted in a more pronounced, 24-fold decrease in binding affinity (Fig. 2*I*). These results clearly demonstrate that the contact between the α-carboxyl moiety of γC and the ADMIDAS metal ion makes an important contribution to affinity and is highly specific both for the carboxyl group and for the chirality of the Val residue.

A free C terminus was not required for headpiece conformational change. At 1 mm (20 × *K_D_*), amidated AGDV increased the hydrodynamic radius from 4.69 to 5.04 nm, whereas at 1 mm (100 × *K_D_*), AGDV increased the radius to 5.2 nm (Fig. 3*A*). Attempts to increase the concentration of AGDV-NH_2_ to 2.5 and 5 mm resulted in headpiece aggregation. However, the use of AGDV at 0.14 mm (16 × *K_D_*) and 0.48 mm (50 × *K_D_*) induced similar shifts in Stokes radius as AGDV-NH_2_ at 1 mm (20 × *K_D_*) (Fig. 3*A*), showing that when corrected for amount of binding, AGDV-NH_2_ and AGDV are similar in ability to induce headpiece opening.

When closed headpiece crystals were soaked with 50 mm AGDV-NH_2_ for 2 h, the peptide was observed in both integrin molecules in the asymmetric unit, with molecule 1 in state 5 (Fig. 4*H*) and molecule 2 in state 3 (Fig. 4*D*). Similar soaking for 2 h with AGDV resulted in binding to headpiece molecule 1 in state 6 and molecule 2 in state 3 (Fig. 4, *G* and *E*). The slight reduction in shape-shifting induced by AGDV-NH_2_ is compatible with its lower affinity. The conformation of AGDV-NH_2_ in state 5 is similar to AGDV in states 6 and 7. The main difference is that the terminal amide oxygen of AGDV-NH_2_ is 3.9 Å away from the ADMIDAS water (Fig. 4*H*), whereas the corresponding distance in AGDV states 6 and 7 are 3.0 and 2.7 Å, respectively (Fig. 4, *G* and *I*). The conformations of Val of AGDV-NH_2_ and AGDV in states 5–8 are very similar (Fig. 4, *G–J*). In state 3, the ADMIDAS metal ion position with AGDV-NH_2_ (Fig. 4*D*) resembled that with RGD (Fig. 4*F*) rather than with AGDV (Fig. 4*E*).

##### A Dicarboxylate α_IIb_β_3_ Antagonist

Some α_IIb_β_3_ antagonists have two carboxyl groups, such as Ro-435054 (22) (see Fig. 7*A*). To test whether the second carboxyl group would orient similarly to the α-carboxyl group of KQAGDV and AGDV, we soaked Ro-435054 into crystals. When soaked in 1 mm Ro-435054 with 2 mm MnCl_2_ and 0.1 mm CaCl_2_, crystals either dissolved, cracked, or diffracted poorly. However, crystals were preserved when we soaked them with 1 mm Ro-435054 for 2 h in Mg^2+^/Ca^2+^. The compound bound to both α_IIb_β_3_ molecules in the asymmetric unit and induced shape-shifting to states 6 and 3 (Table 1). This contrasts with soaking for 24 h with 10 mm GRGDSP in Mg^2+^/Ca^2+^, which only shifted molecule 1 to state 2 and bound to molecule 2 without shifting it from state 1 (4). Density for Ro-435054 was well defined; however, electron densities for ADMIDAS Ca^2+^ ions in both molecules were very weak, and ADMIDAS Ca^2+^ ions were therefore omitted during refinement. Ro-435054 is a peptidomimetic. One of the nitrogens in its N-terminal benzamidine overlaps with the ϵ-amino group of the Lys side chain of KQAGDV. Most importantly, the side chain carboxyl of the Asp residue and the α-carboxyl groups of the Phe residue of Ro-435054 align very well with those of the Asp and Val residues of γC dodecapeptide and AGDV (see Fig. 7*B*).

##### AGDV Induces Integrin Extension on Cell Surfaces Similarly to RGD Peptide and Fibrinogen Dodecapeptide

**FIGURE 5.**
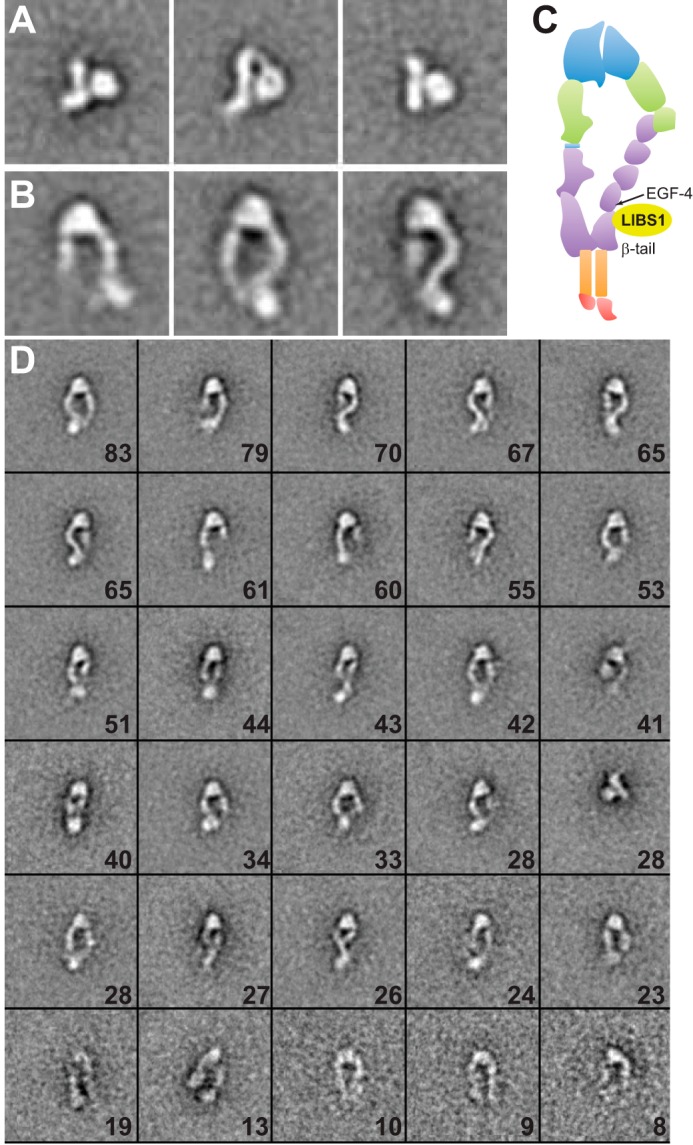
**EM class averages of full-length α_IIb_β_3_.** α_IIb_β_3_ purified from platelets in buffer with dodecylmaltoside (23) was mixed with LIBS1 Fab and subjected to gel filtration and negative stain EM, particle picking, and class averaging as described previously (23). *A*, representative class averages of α_IIb_β_3_ and LIBS1 Fab mixture, showing that LIBS1 does not bind to α_IIb_β_3_ in the closed-bent conformation in 1 mm Mg^2+^ plus 1 mm Ca^2+^. *B*, representative EM class averages of α_IIb_β_3_ bound to a fragment of LIBS1 Fab in the presence of 10 nm L-739,759 and 1 mm Mn^2+^ plus 0.1 mm Ca^2+^. *C*, schematic diagram of integrin domains and approximate binding regions of LIBS1 antibody as shown by EM. *D*, All 30 EM class averages from *B*; each box has a dimension of 120 × 120 pixels with 4.5 Å/pixel. The number of particles in each class average is denoted in each box.

Exposure of LIBS epitopes on integrin α_IIb_β_3_ is widely used as a surrogate of conformational change; however, the type of conformational change (*e.g.* integrin extension or headpiece opening) that LIBS antibodies report on α_IIb_β_3_ is poorly characterized. Detergent-soluble, intact α_IIb_β_3_ particles purified from human platelets are 91% in the bent conformation (23). Incubation with LIBS1 Fab fragment had little effect on the proportion of bent particles (95%), and no binding of LIBS1 Fab was detected by EM (Fig. 5*A*). After incubation with the high-affinity RGD-mimetic L-739758 (24) at a final concentration of 10 nm, plus 1 mm Mn^2+^ and 0.1 mm Ca^2+^, most particles are in extended conformations (23); LIBS1 bound to the lower β-leg of extended α_IIb_β_3_, near the EGF4 and β-tail domains (Fig. 5, *B* and *C*). These results show that LIBS1 exposure does not occur in bent α_IIb_β_3_ and requires extension; because the RGD-mimetic also induces headpiece opening, it remains to be determined whether LIBS1 binding requires headpiece opening in addition to extension.

**FIGURE 6.**
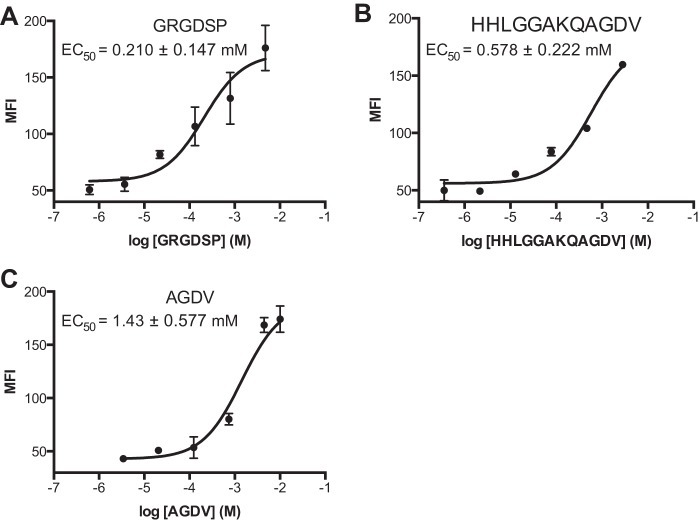
**Peptide ligands induce integrin extension on cell surface.** α_IIb_β_3_ transfectants in the presence of the indicated peptides were stained with LIBS1 antibody, stained with FITC anti-IgG, and subjected to flow cytometry. *MFI* = mean fluorescence intensity. *Error bars* indicate mean ± S.D.

GRGDSP, γC dodecapeptide, and AGDV dose-dependently elicited LIBS1 binding to CHO cells stably expressing full-length human α_IIb_β_3_ (Fig. 6). AGDV induces the same maximal LIBS1 epitope exposure (extension) as GRGDSP and dodecapeptide in 1 mm Mg^2+^ and 1 mm Ca^2+^. However, potencies differ; AGDV is 2.5 times less effective when compared with dodecapeptide (EC_50_ of 1.43 mm *versus* 0.58 mm), and ∼7 times less effective than GRGDSP (EC_50_ = 0.21 mm).

## Discussion

We have shown that the AGDV peptide can bind to α_IIb_β_3_ integrin with affinity comparable with GRGDSP peptide and fibrinogen γC dodecapeptide, and is capable of inducing complete integrin headpiece opening in solution. The structural basis for AGDV-induced headpiece opening was determined by soaking AGDV into crystals and observing a series of intermediate conformational states. AGDV peptides differing in C-terminal modifications showed that the indirect coordination between the α-carboxyl of AGDV and the ADMIDAS metal ion contributes to affinity for α_IIb_β_3_. Binding of AGDV peptide is also able to induce LIBS1 epitope exposure and hence full-length α_IIb_β_3_ integrin extension on cell surfaces.

Crystal structures showed that AGDV has limited contact with the α_IIb_ subunit. Of the total solvent-accessible surface area (25) buried by AGDV on α_IIb_β_3_ (∼340 Å^2^), only 23% (80 Å^2^) is contributed by the α_IIb_ subunit. The α_IIb_ subunit contributes no specific interactions. In contrast, the tetrapeptide has a much larger, 260-Å^2^ interface with β, or 77% of the total buried solvent-accessible surface area. Relative to typical interfaces buried in antibody-protein antigen interactions of 800 Å^2^ (26), the overall burial of AGDV is small. Thus, the highly specific contacts made by AGDV must be important. The interface between AGDV and the integrin βI domain includes multiple hydrogen bonds with the β1-α1 loop, and most importantly, the coordination of Asp side chain carboxyl to the MIDAS metal, and the water-mediated contact with the ADMIDAS metal.

Unlike RGD or γC dodecapeptide, AGDV peptides do not form salt bridges with the integrin α_IIb_ subunit. Despite lacking the salt bridges, Ac-AGDV shows an almost identical affinity with dodecapeptide, and AGDV peptide is only ∼7 times less potent than the dodecapeptide in Mn^2+^/Ca^2+^. These observations show that the contributions in binding free energy of the salt bridge and other additional contacts with α are relatively minor when compared with the interactions with β.

The C-terminal carboxyl group of AGDV contributes positively to binding based on the ∼7-fold reduction in affinity with amidated AGDV (AGDV-NH_2_). Nevertheless, the α-carboxyl group is not required for headpiece opening. In DLS experiments, similar saturation of the headpiece with AGDV-NH_2_ and AGDV gave similar increases in hydrodynamic radius. Moreover, AGDV and AGDV-NH_2_ induced similar headpiece opening when soaked into crystals.

In aggregate, our results show that Asp binding to the β_3_-subunit contributes the majority of the energy for ligand binding and that interaction with the β_3_-subunit is sufficient to induce headpiece opening and extension. These findings also suggest that β_3_ integrins may have broader ligand specificities than previously expected.

The native C-terminal carboxyl of AGDV peptide was more favorable than any other chemical modification we tested, including amidation, methylation, and replacing the C-terminal l-valine with a d-valine. The order of affinity was native carboxyl > amide > methyl ester > d-valine). The C-terminal carboxyl group forms a water-mediated coordination to the ADMIDAS metal (Fig. 4, *G* and *I*) (3). One explanation for the weaker affinity of AGDV-NH_2_ is that the long-range ionic interaction (4.7 Å) between the α-carboxyl anion and ADMIDAS divalent metal cation is absent in AGDV-NH_2_. The other explanation is the different hydrogen bond strength among these derivatives. A charged carboxyl oxygen forms a stronger hydrogen bond to water than an uncharged carbonyl oxygen in an amide or ester. Interestingly, the chirality of Val of AGDV was very important, because substitution with d-valine resulted in a 24-fold reduction in affinity. These results are consistent with the identical orientation and rotamer of the Val side chain in AGDV and AGDV-NH_2_ states 3–7 and in native state 8 of the dodecapeptide. The α-carboxyl group of d-valine cannot form polar contacts with the ADMIDAS, because the side chain of d-valine will clash with Tyr-122 of the β_3_-subunit when its α-carboxyl group is oriented identically to that of l-valine.

**FIGURE 7.**
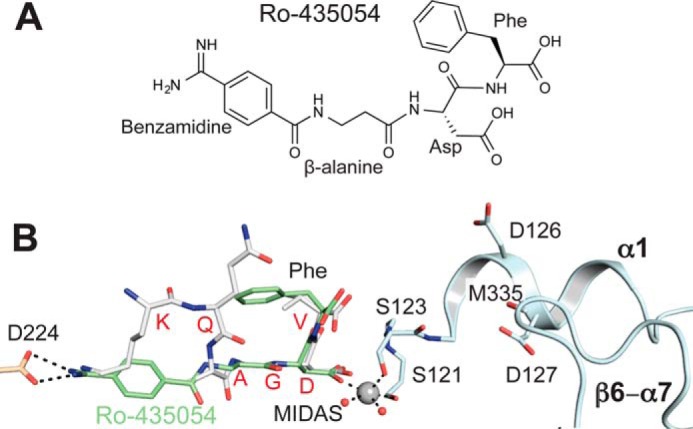
**Structure of Ro-435054 bound to α_IIb_β_3_ headpiece.** *A*, chemical structure of Ro-435054. *B*, overlay of α_IIb_β_3_ complex crystal structures with Ro-435054 (*green carbons*) and γC dodecapeptide (*gray carbons*), after superimposition on main chain atoms of the βI domain. Only α_IIb_β_3_ residues from the Ro-435054 complex are illustrated with carbons colored *wheat* (α_IIb_) or *cyan* (β_3_). The MIDAS Mg^2+^ is shown as a *gray sphere*, and water molecules are shown as *small red spheres. Black dashed lines* represent hydrogen bonds and metal coordinations of the Ro-435054 structure.

The dicarboxylate α_IIb_β_3_ antagonist Ro-435054 binds the high-affinity state of α_IIb_β_3_ ∼100-fold better (*K_D_* = 6 nm) than the low-affinity state (*K_D_* = 580 nm) (27). Ro-435054 is a 4-residue peptidomimetic, with an N-terminal amidinobenzoyl, a β-alanine, an l-Asp, and a C-terminal l-Phe (Fig. 7*A*). Most interestingly, the C-terminal Phe residue of Ro-435054 adopts an identical orientation to the Val of KQAGDV and AGDV and thus may be considered a fibrinogen-mimetic. The high-affinity RGDF peptide (19) may be considered both fibrinogen-mimetic and RGD-mimetic. RGDF has the same two C-terminal amino acids as Ro-435054 and may be predicted to bind similarly.

Following fibrin formation in hemostasis, fibrin molecules are cross-linked by factor XIIIa. Factor XIIIa catalyzes the transglutamination reaction between Lys-406 of γC in one fibrin molecule and Gln-398 or Gln-399 in another to cross-link two neighboring dimers (28). Gln-399 immediately precedes the γC dodecapeptide, and in the dodecapeptide, Lys-406 is the Lys of KQAGDV that binds to α_IIb._ This C-terminal portion of the fibrinogen γC-subunit is unstructured in the absence of binding to α_IIb_β_3._ Although factor XIIIa covalently stabilizes fibrin, it at the same time makes Lys-406 unavailable for integrin binding. Because our results demonstrate that this Lys is not required for binding to α_IIb_β_3_, it is conceivable that the cross-linked fibrin may still interact with α_IIb_β_3_ using the C-terminal AGDV sequence. Further investigations on α_IIb_β_3_ binding to factor XIIIa-cross-linked fibrin are needed to understand the physiological relationship between fibrin cross-linking and binding to α_IIb_β_3_.

## Author Contributions

F.-Y. L., J. Z., and E. T. E contributed to research design, carried out experiments, analyzed data, and wrote the manuscript. N. E. H. analyzed the data and helped prepare the manuscript. T. A. S. conceived the experimental design, analyzed the data, and wrote the manuscript.

## Footnotes

3 The abbreviations used are:

MIDAS: metal ion-dependent adhesion site
ADMIDAS: adjacent to MIDAS
DLS: dynamic light scattering
LIBS: ligand-induced binding site
Ac-AGDV: *N*-acetylated AGDV.
